# Genetic dependency of Alzheimer’s disease-associated genes across cells and tissue types

**DOI:** 10.1038/s41598-021-91713-2

**Published:** 2021-06-08

**Authors:** Suraj K. Jaladanki, Abdulkadir Elmas, Gabriel Santos Malave, Kuan-lin Huang

**Affiliations:** Department of Genetics and Genomic Sciences, Center for Transformative Disease Modeling, Tisch Cancer Institute, Icahn Institute for Data Science and Genomic Technology, Icahn School of Medicine at Mount Sinai, New York, NY 10029 USA

**Keywords:** Cancer genomics, Drug screening, Target identification, Neurological disorders

## Abstract

Effective treatments targeting disease etiology are urgently needed for Alzheimer’s disease (AD). Although candidate AD genes have been identified and altering their levels may serve as therapeutic strategies, the consequence of such alterations remain largely unknown. Herein, we analyzed CRISPR knockout/RNAi knockdown screen data for over 700 cell lines and evaluated cellular dependencies of 104 AD-associated genes previously identified by genome-wide association studies (GWAS) and gene expression network studies. Multiple genes showed widespread cell dependencies across tissue lineages, suggesting their inhibition may yield off-target effects. Meanwhile, several genes including *SPI1*, *MEF2C*, *GAB2*, *ABCC11*, *ATCG1* were identified as genes of interest since their genetic knockouts specifically affected high-expressing cells whose tissue lineages are relevant to cell types found in AD. Overall, analyses of genetic screen data identified AD-associated genes whose knockout or knockdown selectively affected cell lines of relevant tissue lineages, prioritizing targets for potential AD treatments.

## Introduction

Globally, an estimated 43.8 million individuals were affected by dementia in 2016, a more than two-fold increase from 20.2 million in 1990, and is expected to continue rising^1^. Alzheimer’s disease (AD) is the leading cause of dementia, and only supportive treatments are currently available for AD. AD medications consist of cholinesterase inhibitors (donepezil, galantamine, and rivastigmine), NMDA receptor uncompetitive antagonist memantine, or a combination of both (donepezil)^2^. These medications only address symptoms and are helpful for a limited timespan in improving patients’ cognitive abilities. They are not disease-modifying therapies and do not alter disease progression. Other novel therapies for both early-onset AD (EOAD) and late-onset AD (LOAD) have focused on *APP*, *PSEN1*, and *PSEN2,* identified through rare mutations in familial EOAD cases*,* that cause amyloid-β deposition. Testing has been carried out through the administration of anti-amyloid-β exogenous monoclonal antibodies, but this approach has not yielded promising results^3–5^. One such antibody is aducanumab which was recently approved for expedited review by the FDA but is yet to be validated and demonstrates inconclusive clinical efficacy^6,7^. Several gamma secretase inhibitors have also been tested but failed in clinical studies and were ultimately discontinued^5^.

Recent research has discovered new genetic risk loci for LOAD by genome-wide association studies (GWAS) or transcriptomic studies. Identified genes have been found to be strongly expressed in immunity, lipid metabolism, tau binding proteins, and amyloid precursor protein metabolism^8^. One recent study confirmed 20 previous risk loci (*CR1*, *BIN1*, *INPP5D*, *HLA-DRB1*, *TREM2*, *CD2AP*, *NYAP1*, *EPHA1*, *PTK2B*, *CLU*, *SPI1*, *MS4A2*, *PICALM*, *SORL1*, *FERMT2*) and identified five novel loci (*IQCK*, *ACE*, *ADAM10*, *ADAMTS1,* and *WWOX*)^8^. A parallel GWAS study found 29 risk loci, including previously classified risk loci and several novel loci^9^. In parallel, transcriptomic analyses found at least 50 network hub genes upregulated in co-expression networks of LOAD compared to normal brain tissues. For example, *TYROBP* was identified as a pivotal regulator of the phagocytotic pathway^10^, cross-validating with immunity-implicated genes found by GWAS.

While GWAS and transcriptomic studies have nominated gene candidates associated with AD pathology, the therapeutic viability of manipulating functions or levels of such targets remain largely unknown. An ideal gene target should not only show sensitivity by being implicated in AD etiology, but also specificity by which its targeting intervention would not cause widespread, off-target effects (e.g., house-keeping genes or genes serving essential cognitive functions). Genome-wide CRISPR-Cas9 knockout or RNAi knockdown screens are tools which can help prioritize therapeutic targets and can guide inhibition treatments that are both effective and potentially have reduced toxicity^11^. The Cancer Dependency Map Project (DepMap) has curated dependency profiles of almost 18,000 genes across more than 700 human cell lines^11,12^. While involving multiple cell lines that share lineages with cells implicated in AD, the dataset has not been used to evaluate AD targets. Herein, we leveraged the large-scale transcriptomic/proteomic expression and dependency data from the DepMap genome-scale CRISPR-Cas9 and RNAi screens to assess the effects of knocking out and knocking down candidate AD associated genes, revealing their potential as precision therapeutic targets.

## Methods

### Data source and download

Genes used in this publication were obtained from multiple papers. Genome wide associated studies (GWAS) that identified genes implicated in AD were downloaded from Kunkle et al. and Jansen et al.^8,9^. 42 unique genes from these two sources were compiled, and *APP*, *PSEN1*, and *PSEN2* were added to the AD risk gene list^13^. 60 key AD network hub genes associated with AD were downloaded from Zhang et al.^10^. Cancer gene expression and dependency scores were downloaded from the DepMap Portal Public 20Q2 release in May 2020^14^.

### Expression-driven dependency using DepMap

The DepMap Public 20Q2 release contains the Achilles dataset and results of genome-scale CRISPR knockout screens for 19,144 genes across 1206 cell lines, including both cancer and normal cell lines. 20 tissue types from DepMap with at least 25 cell lines each were selected for further analysis from an original list of 40 tissue types. For each of the 104 genes associated with AD, gene expressions and corresponding CERES dependency scores were analyzed. CERES is a computational method developed by Meyers et al., which estimates gene dependency levels derived from CRISPR-Cas9 essentiality screens and factors in the possibility of an increase in false positives in copy number amplified locations^15^. For each AD-associated gene, we calculated the proportion of significantly negative CERES scores (< − 0.5) by tissue type to generate heatmaps. A negative CERES score indicates that a gene knockout results in a slower growth rate of a cell line, and a score lower than − 0.5 indicates a notable reduction^15^. We also calculated the Pearson correlation and corresponding p-values for each AD-associated gene after stratifying the gene expression and CERES scores by tissue type. The analysis was limited to examining expression-driven dependencies in three tissue types: hematopoietic and lymphoid tissue, central nervous system, and autonomic ganglia for their involvement in AD presentation and progression^16–18^. Overall, 50 gene and tissue combinations were significant at the p-value of 0.05. Volcano plots display the correlation coefficients (x-axis) against the negative log of FDR corrected p-values (y-axis). We focused on gene-tissue combinations with negative correlation coefficients as a high expression of the AD-associated gene and corresponding low CERES score indicates that the gene is needed for cancer cell survival in knockout experiments. Scatterplots of dependency versus expression were plotted for AD gene tissue combinations with the highest negative log p-values, and correlation coefficients were calculated.

The DepMap Public 20Q2 release also contains genetic dependency scores estimated from RNAi and CRISPR loss-of-function screens for 17,309 genes across 712 cell lines^14^. These scores were calculated using DEMETER2, a framework developed by McFarland et al. to unify results from multiple large-scale RNAi screening datasets^19^. Calculations of negative dependency scores, volcano plots highlighting correlations, and scatterplots of AD-associated genes were completed in a similar process as CERES scores. Analysis of genetic dependency data was performed using Python (v 3.7.6), Numpy (v 1.7.4), and SciPy (v 1.3.2).

## Results

### Gene list and DepMap data assembly

A curated list of 104 AD-associated genes, consisting of 44 AD risk genes and 60 AD network hub genes, were utilized for this study (see Supplementary Table S1). There were 20 tissue types with data for at least 25 cell lines each from the DepMap 2020 Q2 release which were selected for further analysis (Table 1). We specifically highlighted results from three tissue types most relevant to AD etiology: hematopoietic and lymphoid tissue (HL, given the now established role of immune response and phagocytotic pathway in AD), central nervous system (CNS), and autonomic ganglia (AG)^16–18^.

**Table 1:** Overview of tissue types and number of unique cell lines used for cell line genetic dependency analysis.

Lineage	Dependency CERES	Dependency DEMETER2	mRNA
Autonomic ganglia	20	9	28
Biliary tract	28	1	39
Bone	28	15	37
Breast	34	82	57
Central nervous system	60	55	84
Endometrium	24	20	33
Haematopoietic and lymphoid tissue	82	61	205
Kidney	21	29	34
Large intestine	37	44	67
Liver	23	18	25
Lung	97	129	192
Oesophagus	25	24	32
Ovary	42	38	58
Pancreas	34	33	51
Pleura	9	6	17
Skin	51	46	67
Soft tissue	38	25	65
Stomach	26	25	41
Upper aerodigestive tract	31	18	43
Urinary tract	29	12	36

20 tissue types from the Cancer Dependency Map (DepMap) project with data for at least 25 cell lines were utilized for this analysis.

### Pan-tissue genetic dependency

**Figure 1:**
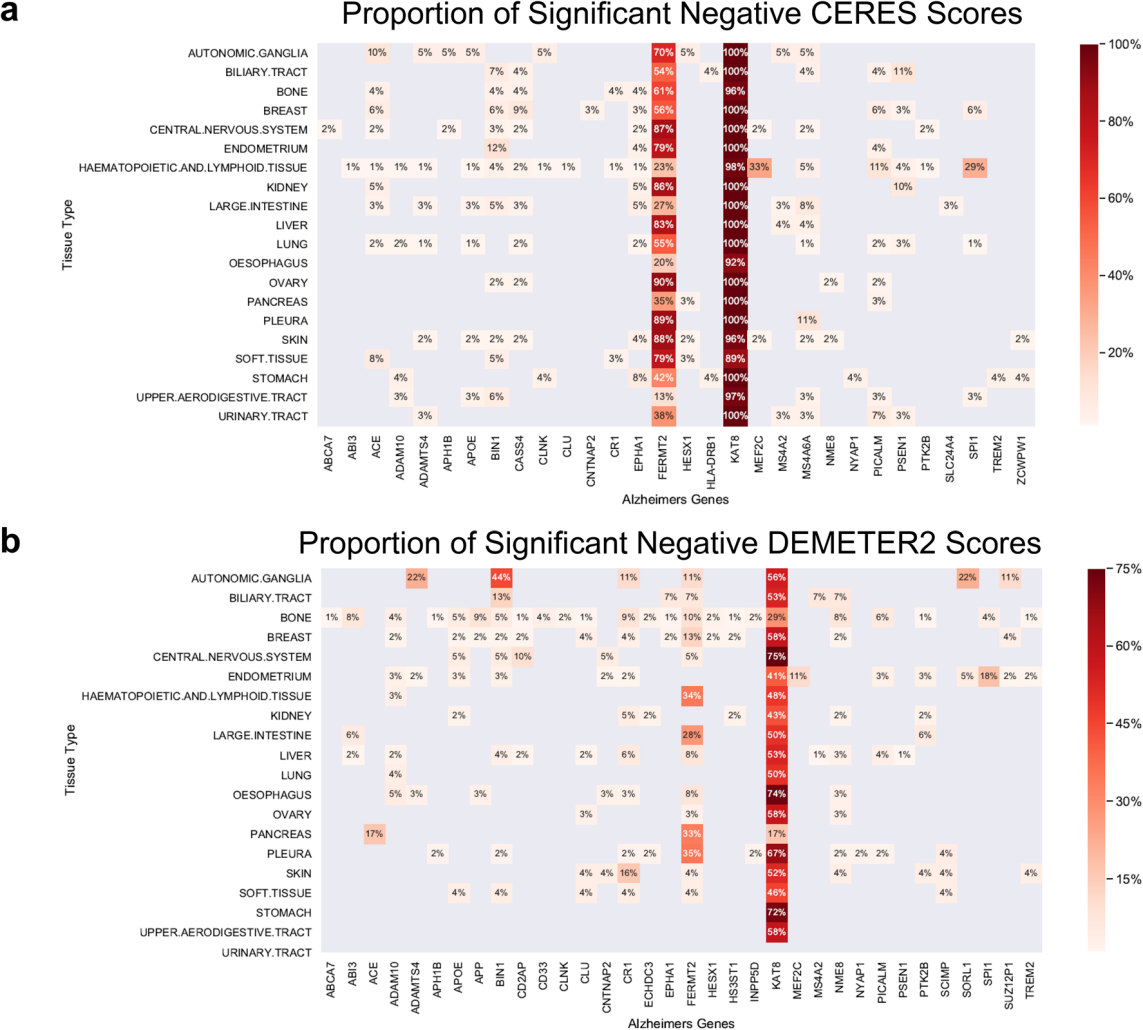
Genetic dependency of AD risk genes in CRISPR knockout and knockdown screens across tissue lineages. (**a**) The proportion of pass-DepMap-threshold negative dependency scores (< − 0.5) in cell lines by tissue types based on the CERES scores derived from CRISPR knockout screens. (**b**) The proportion of pass-DepMap-threshold negative dependency scores (< − 0.5) in cell lines by tissue types based on the DEMETER2 scores derived from RNAi knockdown screens. For both (**a**) and (**b**), 20 tissue types from the Cancer Dependency Map (DepMap) project with data for at least 25 cell lines were utilized to evaluate 44 AD risk genes. A negative dependency score indicates a gene’s necessity for a cell line’s growth and survival.

To examine the genetic dependency of AD-associated genes across tissues, we identified the proportion of cell lines within each tissue type showing significant negative dependency scores (< − 0.5), as lower scores indicate that a gene is required for cell survival and proliferation. From 44 AD risk genes found through GWAS, *KAT8* showed predominant negative dependency scores in the CRISPR knockout data (CERES score (< − 0.5), with nearly 100% negative dependency scores across all 22 examined tissue types (Fig. 1a). *FERMT2* had varying levels of negative CERES scores across tissues with ovary, pleura, and skin cell lines having the highest proportion of negative scores. *MEF2C* and *SPI1* have negative CERES scores localized to the hematopoietic and lymphoid tissue (HL) cell lineage, with 33% and 29%, respectively. We also identified the proportion of cell lines with significant negative genetic dependency scores (DEMETER2 score < − 0.5) utilizing data from large-scale RNAi screens. From the list of AD risk genes, *KAT8* had a range of significant negative DEMETER2 scores from 17 to 75% across tissue types, with a peak of 75% in endometrium cell lines (Fig. 1b). *ADAMTS4, BIN1, SORL1,* and *SUZ12P1* had their highest levels of negative DEMETER2 scores in autonomic ganglia (AG) cell lines. *SPI1* had its highest proportion of negative DEMETER2 scores in HL cell lines.

**Figure 2:**
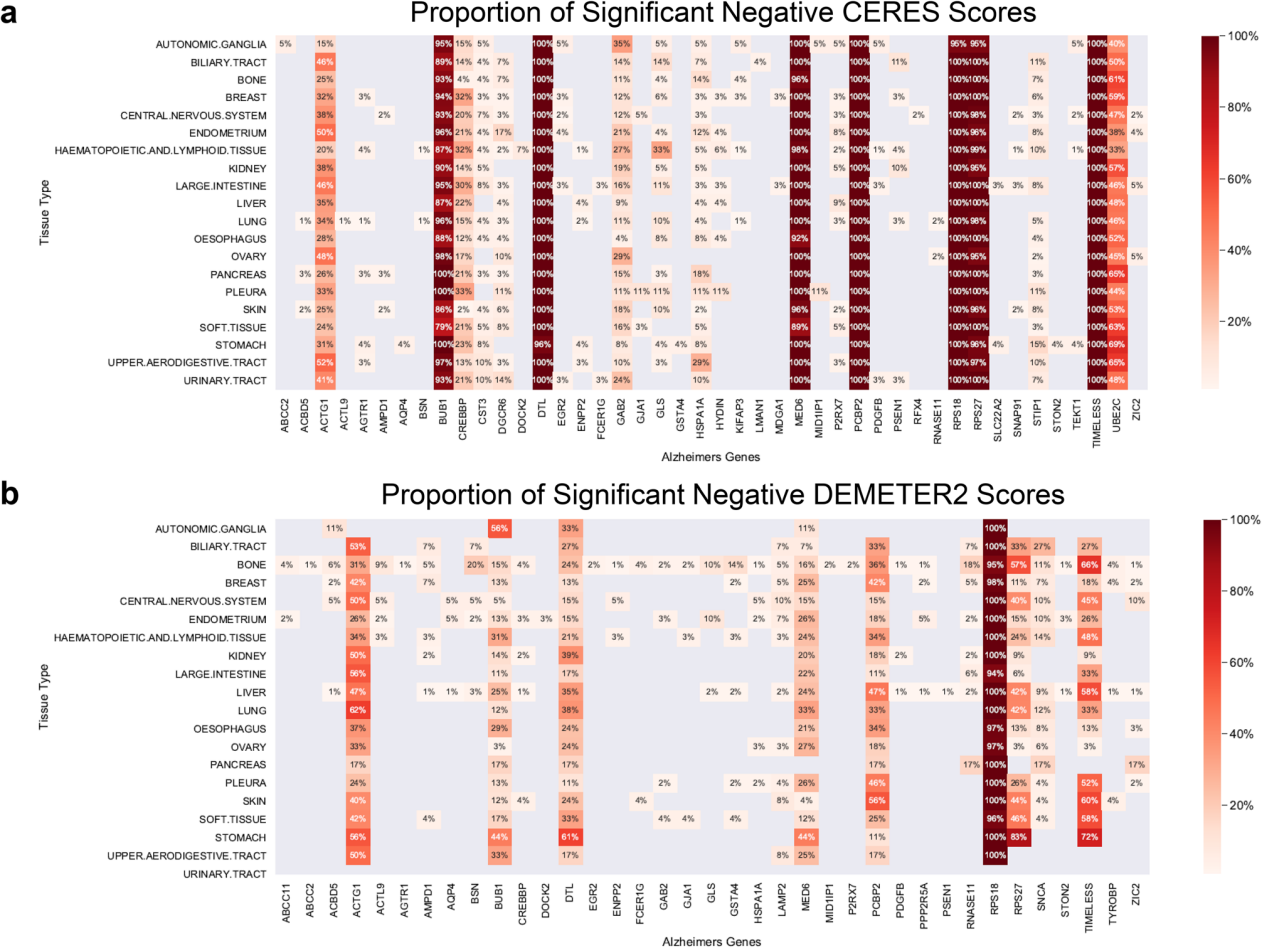
Genetic dependency of AD network hub genes in CRISPR knockout and knockdown screens across tissue lineages. (**a**) The proportion of pass-DepMap-threshold negative dependency scores (< − 0.5) in cell lines by tissue types based on the CERES scores derived from CRISPR knockout screens. (**b**) The proportion of pass-DepMap-threshold negative dependency scores (< − 0.5) in cell lines by tissue types based on the DEMETER2 scores derived from RNAi knockdown screens. For both (**a**) and (**b**), 20 tissue types from the Cancer Dependency Map (DepMap) project with data for at least 25 cell lines were utilized to evaluate 60 AD network hub genes. A negative dependency score indicates a gene’s necessity for a cell line’s growth and survival.

Among the 60 AD network hub genes, *BUB1, DTL, MED6, PCBP2, RPS18, RPS27,* and *TIMELESS* had significant negative CERES scores of approximately 100% across tissue types (Fig. 2a). *UBE2C, ACTG1, CREBBP* had varying levels of negative CERES scores ranging from 20 to 60% across tissue types. *GLS* and *GAB2* showed highest proportion of negative CERES scores in HL (33%) and AG (35%) cell lines, respectively. From AD-associated network hub genes, *RPS18* had approximately 100% negative DEMETER2 scores across all tissue types (Fig. 2b). *ACTG1, BUB1, DTL, MED6, PCBP2, RPS27,* and *TIMELESS* had varying levels of negative DEMETER2 scores from 10 to 75% across tissue types. The largely concordant CRISPR/RNAi screen results suggest that knocking out/down of several genes, including *KAT8* and *FERM2*, results in widespread consequences affecting most cells’ survival, whereas other candidates (e.g., *MEF2C* in HL, *GAB* in AG) more selectively affect fractions of AD-relevant cell types, thus may serve as better targets.

### Expression-driven cellular dependencies of AD risk genes

**Figure 3:**
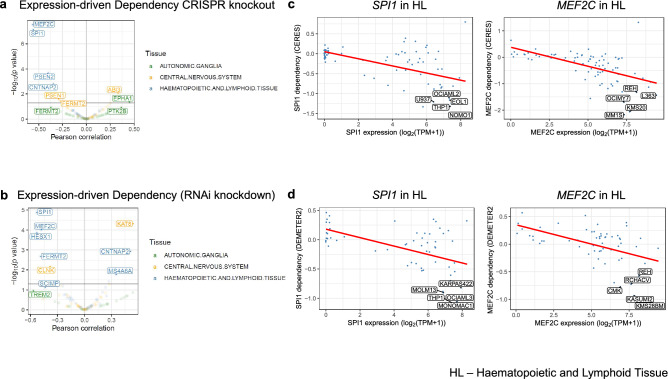
Expression-driven Dependency of AD Risk Genes. (**a**) A volcano plot showing expression-driven dependency of AD risk genes based on CRISPR knockout screen data. (**b**) A volcano plot showing expression-driven dependency of AD risk genes based on RNAi knockdown screen data. For (**a**) and (**b**), Pearson correlation coefficients were calculated for AD risk genes using a gene’s expression versus the dependency scores for three tissue types (color-coded) relevant to AD etiology: hematopoietic and lymphoid tissue (HL), central nervous system (CNS), and autonomic ganglia (AG). The correlation plots of the expression-driven dependency were shown for associations found in the HL cell lines, including (**c**) *SPI1* and *MEF2C* expression with their respective CERES dependency scores, as well as (**d**) with their respective DEMETER2 dependency scores. The best-fitted regression lines are shown in red and the cell lines with the highest expression and dependency are further labeled in each plot.

We reasoned that AD-associated genes may show aberrant expression in disease-driving/affected cells. Thus, the candidate genes would likely serve as better targets if their knockout or knockdown most strongly influenced the cells showing aberrant expression of the targeted genes. We next sought to further filter for genes whose expression is significantly correlated with dependencies of cell lines within these tissue types, i.e., expression-driven dependency. We conducted a systematic Pearson correlation analysis to identify such genes of interest, and identified four genes whose expression was significantly associated with cellular dependencies (FDR < 0.05), including *MEF2C* in HL cell lines (R = − 0.6, FDR = 8.13e−06), *SPI1* in HL cell lines (R = − 0.6, FDR = 9.38e−06), *PSEN2* in HL cell lines (R = − 0.4, FDR = 0.0131), and *CNTNAP2* in HL cell lines (R = − 0.3, FDR = 0.0433) (Fig. 3a). We further conducted the correlation analysis using RNAi knockdown-based DEMETER2 scores to identify similarities and differences to CERES results. We found five genes with significant expression-driven dependency (FDR < 0.05), including *SPI1* in HL cell lines (R = − 0.5, FDR = 0.00147), *MEF2C* in HL cell lines (R = − 0.5, FDR = 0.00545), *HESX1* in HL cell lines (R = − 0.5, FDR = 0.00578), *CNTNAP2* in HL cell lines (R = 0.5, FDR = 0.0424), *KAT8* in CNS cell lines (R = 0.5, FDR = 0.00348) **(**Fig. 3b).

We next highlighted the cell lines that were most affected by knockout/knockdowns; given the challenge of functionally modeling AD in human systems^20^, these lines may provide alternatives that show aberrantly high expression of the selected AD-associated genes. For *SPI1* in HL cell lines, these include NOMO1, THP1, MONOMAC1, THP1, and EOL1. For *MEF2C* in HL cell lines, these were MM1S, KMS20, OCIMY7, KMS28BM, KASUMI2, and L363 (Fig. 3c,d).

### Expression-driven cellular dependencies of AD network hub genes

**Figure 4:**
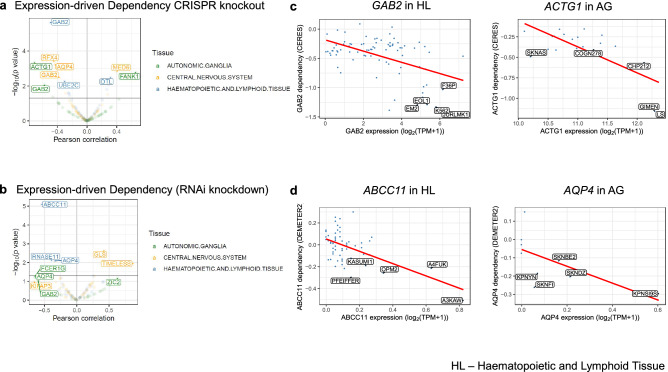
Expression-driven Dependency of AD Network Hub Genes. (**a**) A volcano plot showing expression-driven dependency of AD network hub genes based on CRISPR knockout screen data. (**b**) A volcano plot showing expression-driven dependency of AD network hub genes based on RNAi knockdown screen data. For (**a**) and (**b**), Pearson correlation coefficients were calculated for AD risk genes using a gene’s expression versus the dependency scores for three tissue types (color-coded) relevant to AD etiology: hematopoietic and lymphoid tissue (HL), central nervous system (CNS), and autonomic ganglia (AG). The correlation plots of the expression-driven dependency were shown for associations found for (**c**) *GAB2* in HL cell lines and *ACTG1* in AG cell lines against CERES scores (**d**) *ABCC11* in HL cell lines and *AQP4* in AG cell lines against DEMETER2 scores. The best-fitted regression lines are shown in red and the cell lines with the highest expression and dependency are further labeled in each plot.

We applied the same expression-driven dependency analysis for the AD network hub genes. Based on the CRISPR screen data, we identified six genes whose expression was significantly associated with cellular dependencies (FDR < 0.05), including *GAB2* in HL cell lines (R = − 0.5, FDR = 0.000221), *ACTG1* in AG cell lines (R = − 0.7, FDR = 0.0203), RFX4 in CNS cell lines (R = − 0.4, FDR = 0.0203), *AQP4* in CNS cell lines (R = − 0.4, FDR = 0.0222), *FANK1* in AG cell lines (R = 0.7, FDR = 0.0436), *MED6* in CNS cell lines (R = 0.4, FDR = 0.043254) (Fig. 4a). Using the RNAi screen data, we identified one gene with significant expression-driven dependency (FDR < 0.05), *ABCC11* in HL cell lines (R = − 0.5, FDR = 0.00147) (Fig. 4b).

To highlight potential cell lines that can help study the implication of targeting aberrant expressions of these genes, JURLMK1 showed the lowest CERES dependency score and high expression for *GAB2* in HL cell lines (Fig. 4c). For *ACTG1* in AG cell lines, the cell lines of interest were LS, GIMEN, CHP212, SKNAS, and COGN278. Based on DEMETER dependency scores, for *ABCC11* in HL cell lines, A3KAW, A4FUK, OPM2, KASUMI1, and PFEIFFER were highlighted (Fig. 4d). For *AQP4* in AG, we noted that the cell lines KPNSI9S, SKNFI, SKNDZ, KPNYN, and SKNBE2 show expression. For *GLS* in CNS, the cell lines were LN235, U178, KNS60, LN215, and HS683 (see Supplementary Fig. S1).

## Discussion

We conducted a comprehensive genetic dependency analysis of 104 AD-associated genes using expression and genetic screen data from over 700 cell lines. The analysis enabled us to identify whether genetic knockdown or knockout of these AD-associated genes may affect cellular survival. Our results show that the knockdown or knockout of multiple AD-associated genes (*KAT8*, *FERMT2*, *BUB1*, *DTL*, *MED6*, *PCBP2*, *RPS18*, *RPS27*, *TIMELESS*, *ACTG1*, and *UBE2C*) significantly reduced survival of cell lines across multiple tissues. Thus, down-regulating or inhibiting these genes could lead to pervasive damaging effects and may have limited therapeutic viability.

Meanwhile, several genes showed dependency that is correlated with gene expression within cell lineages. After limiting our analysis to three tissue types implicated in AD, hematopoietic and lymphoid tissue, central nervous system, and autonomic ganglia, we demonstrated that *SPI1*,* MEF2C*, *GAB2*, and *ABCC11* showed expression-driven dependencies in hematopoietic and lymphoid tissue cell lines. *SPI1* and *MEF2C* were consistently identified in both CRISPR knockout and RNAi screen data, further highlighting their potential as AD treatment targets.

Dysregulated microglial response is a hallmark of AD^21,22^, and the candidates showing expression-driven dependency in the hematopoietic and lymphoid tissue analysis may present targets whose intervention may only affect disease-associated microglia (DAM). For example, SPI1 (PU.1) is a transcription factor encoded by *SPI1*; it is considered as a master regulator of macrophages necessary for the development and differentiation of microglia. GWAS, eQTL, and epigenetic analyses have recently implicated *SPI1* and its regulated network in AD^23^. SPI1 specifies regulatory regions and establishes the chromatin landscape^24^, and its target genes include *TYROBP*, a key regulator upregulated in LOAD^10^. In a study by Olmos-Alonso, et al., inhibition of Colony Stimulating Factor 1 Receptor (*Csf1r*), another target gene of SPI1, led to decreased expression of SPI1 and microglial proliferation in APPswe/PSEN1dE9 mice, a transgenic model of AD-like pathology^25^. As another example, Myocyte-enhancer factor 2C (*MEF2C*) has been identified as a candidate regulator in establishing the chromatin landscape of microglia^24^. *MEF2C* restrains microglial inflammatory response^26^, which is associated with AD pathology in multiple recent studies, and its expression is lost in aging brains. The expression of *MEF2C* in microglia is negatively regulated by interferon type I (IFN-1) expressed chronically during aging^14^. Chronically expressed IFN-1 downregulates *MEF2C,* and loss of *MEF2C* leads to a ‘priming’ state as microglia become more sensitive to immune stimuli. A study by Sao et al. demonstrated that *MEF2C* mRNA expression in AD subjects was significantly lower than expression in control subjects^27^. Given that AD is a neurodegenerative disease with a chronic pro-inflammatory state, downregulation of *MEF2C* expression may contribute to exaggerated pro-inflammatory conditions of the disease^27^. Our results support the importance of *SPI1* and *MEF2C* in HL cells expressing the respective genes (Fig. 3). While these genes may be inhibited or augmented in a targeted manner, the effects of various dosing and timing needs to be thoroughly examined due to their regulatory functions.

A limitation of our study is that most cell lines screened are not derived from AD-patient or brain tissues. Cell lines in the DepMap project, as most of the currently established human cell lines, are derived from cancer samples that may show properties not relevant to AD etiologies. Further, our analysis may miss insights from several critical cell types in AD not represented in DepMap, such as astrocytes and oligodendrocytes, and results for microglia are approximated using hematopoietic and lymphoid cell data. A wide array of cell lines derived from such tissues is unlikely to be available soon. Meanwhile, the gene candidates nominated herein to affect proximal cell types may be further investigated using relevant models, such as patient-derived iPSC or further derived cell lines. Data from genetic screen strategies targeting AD etiology could also strengthen findings. For example, Chiu et al. (2020) used a CRISPR-Cas9 screen to identify regulators of amyloid β peptide (Aβ) synthesis and described *Cib1* as a novel negative regulator in this process^28^. Lastly, our expression-driven dependency analysis identifies targets whose knockout/knockdown/inhibition shows specificity in disease-aberrant cells, and even with limited use of AD models, our findings help rule out genes that are essential for cell lines across tissues or show unpredictable essentiality patterns that may be undesirable for a treatment target.

## Conclusions

This study utilizes CRISPR knockout/RNAi knockdown screen data from DepMap to highlight potential candidates, from a list of AD-associated genes, to be further investigated as AD treatment targets. The expression-driven dependency analyses uniquely identify *SPI1*, *MEF2C*, *GAB2*, *ABCC11*, and *ACTG1*, to have desirable on- vs. off-target effects. These results provide a basis for applying these methods to newly identified genes in AD and evaluating targets in other genetic disorders.

## Supplementary Information

Supplementary Information.

## Data Availability

The datasets generated and analyzed in the current study are available from the DepMap project under the DepMap Public 20Q2 release https://doi.org/10.6084/m9.figshare.12280541.v3(14).

## Abbreviations

AD: Alzheimers’ disease
GWAS: Genome-wide association studies
DepMap: The Cancer Dependency Map Project
EOAD: Early-onset Alzheimer’s disease
LOAD: Late-onset Alzheimer’s disease
Aβ: Amyloid β peptide
HL: Hematopoietic and lymphoid tissue
CNS: Central nervous system
AG: Autonomic ganglia
