# Supplementary material for: Genetic dependency of Alzheimer’s disease-associated genes across cells and tissue types
Source: Sci Rep. 2021 Jun 8;11:12107. doi: 10.1038/s41598-021-91713-2 (PMC8187448; doi:10.1038/s41598-021-91713-2)
Supplement: Supplementary file 1 — Supplementary Information. [file 41598_2021_91713_MOESM1_ESM.pdf]

Genetic Dependency of Alzheimer's Disease-Associated Genes across Cells and Tissue Types

Suraj K Jaladanki[1#], Abdulkadir Elmas[1#], Gabriel Santos Malave[1], Kuan-lin Huang[1*]

[1]Department of Genetics and Genomic Sciences, Center for Transformative Disease Modeling, Tisch Cancer Institute, Icahn Institute for Data Science and Genomic Technology, Icahn School of Medicine at Mount Sinai, New York, NY 10029, United States.

[#]These authors contributed equally.

*Corresponding Author:
Kuan-lin Huang, Ph.D.
Department of Genetics and Genomic Sciences
Icahn School of Medicine at Mount Sinai
New York, NY 10029
Email: kuan-lin.huang@mssm.edu

Supplementary Information

Supplementary Table S1

Title: Full AD-Associated Gene List Utilized in Study

Description: The gene names for the 104 AD-associated genes utilized in the study are provided in alphabetical order.

Supplementary Figure S1

Title: *GLS* in CNS cell lines against DEMETER2 scores

Description: *GLS* was identified in Figure 2b as a gene showing expression-driven dependency in CNS cell lines from RNAi knockdown screen data. The correlation plots of expression-driven dependency is shown for *GLS* in CNS cell lines against DEMETER2 scores.

Supplementary Table S1: Full AD-Associated Gene List Utilized in Study

ABCA7	BUB1	FANK1	MED6	RFX4	TYROBP
ABCC11	BZRAP1-AS1	FCER1G	MEF2C	RNASE11	UBE2C
ABCC2	C1ORF143	FERMT2	MID1IP1	RPS18	ZCWPW1
ABI3	CASS4	GAB2	MLL3S	RPS27	ZIC2
AC074212.3	CD2AP	GJA1	MS4A2	SCIMP	
ACBD5	CD33	GLS	MS4A6A	SFRS3	
ACE	CLNK	GSTA4	MT1JP	SLC22A2	
ACTG1	CLU	HESX1	MT1M	SLC24A4	
ACTL9	CNTNAP2	HLA -DRB1	MT1P3	SNAP91	
ADAM10	CR1	HLA-DRB1	NME8	SNCA	
ADAMTS4	CREBBP	HS3ST1	NYAP1	SORL1	
AGTR1	CST3	HSPA1A	P2RX7	SPI1	
ALPK2	DGCR6	HYDIN	PCBP2	STATIP1	
AMPD1	DOCK2	INPP5D	PCTK2	STIP1	
APH1B	DOPEY1	INPPD5	PDGFB	STON2	
APOE	DTL	KAT8	PICALM	SUZ12P1	
APP	ECHDC3	KIFAP3	PPP2R5A	TEKT1	
AQP4	EGR2	LAMP2	PSEN1	TIMELESS	
BIN1	ENPP2	LMAN1	PSEN2	TREM2	
BSN	EPHA1	MDGA1	PTK2B	TUBB4	

The gene names for the 104 AD-associated genes utilized in the study are provided in alphabetical order.

Supplementary Figure S1: *GLS* in CNS cell lines against DEMETER2 scores

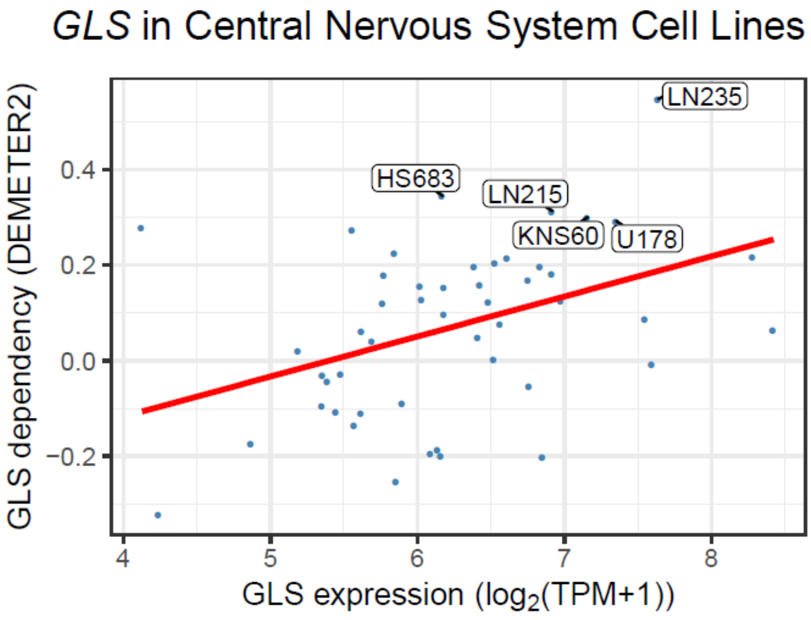

Description: *GLS* was identified in Fig. 2b as a gene showing expression-driven dependency in CNS cell lines from RNAi knockdown screen data. The correlation plot of expression-driven dependency is shown for *GLS* in CNS cell lines against DEMETER2 scores.
